# Supplementary material for: Unveiling the Bioactive Potential of the Invasive Jellyfish Phyllorhiza punctata Through Integrative Transcriptomic and Proteomic Analyses
Source: Biomolecules. 2025 Aug 4;15(8):1121. doi: 10.3390/biom15081121 (PMC12383608; doi:10.3390/biom15081121)
Supplement: Supplementary file 1 [file biomolecules-15-01121-s001.zip › Suplementary Material/FileS3/PEPFOLD4/XP_065064938_11926/clusters.html]

 PEP-FOLD clusters


Download archive of all models  here   

|  |  |  |
| --- | --- | --- |
| # | file | sOPEP |
| # cluster 1 - Size: 26 |  |  |
|  | XP\_065064938\_11926-model1 | -13.431 |
|  | XP\_065064938\_11926-model1.1 | -13.431 |
|  | XP\_065064938\_11926-model1.2 | -13.431 |
|  | XP\_065064938\_11926-model1.3 | -13.431 |
|  | XP\_065064938\_11926-model1.4 | -13.431 |
|  | XP\_065064938\_11926-model1.5 | -13.431 |
|  | XP\_065064938\_11926-model1.6 | -13.431 |
|  | XP\_065064938\_11926-model1.7 | -13.431 |
|  | XP\_065064938\_11926-model1.8 | -13.431 |
|  | XP\_065064938\_11926-model1.9 | -13.381 |
|  | XP\_065064938\_11926-model1.10 | -13.381 |
|  | XP\_065064938\_11926-model1.11 | -13.381 |
|  | XP\_065064938\_11926-model1.12 | -13.381 |
|  | XP\_065064938\_11926-model1.13 | -13.381 |
|  | XP\_065064938\_11926-model1.14 | -13.381 |
|  | XP\_065064938\_11926-model1.15 | -13.381 |
|  | XP\_065064938\_11926-model1.16 | -13.381 |
|  | XP\_065064938\_11926-model1.17 | -13.381 |
|  | XP\_065064938\_11926-model1.18 | -13.381 |
|  | XP\_065064938\_11926-model1.19 | -13.2437 |
|  | XP\_065064938\_11926-model1.20 | -13.2437 |
|  | XP\_065064938\_11926-model1.21 | -13.2437 |
|  | XP\_065064938\_11926-model1.22 | -13.2437 |
|  | XP\_065064938\_11926-model1.23 | -13.1859 |
|  | XP\_065064938\_11926-model1.24 | -13.1859 |
|  | XP\_065064938\_11926-model1.25 | -13.1859 |
| # cluster 2 - Size: 11 |  |  |
|  | XP\_065064938\_11926-model2 | -13.4112 |
|  | XP\_065064938\_11926-model2.1 | -13.4112 |
|  | XP\_065064938\_11926-model2.2 | -13.4112 |
|  | XP\_065064938\_11926-model2.3 | -13.3485 |
|  | XP\_065064938\_11926-model2.4 | -13.3485 |
|  | XP\_065064938\_11926-model2.5 | -13.3485 |
|  | XP\_065064938\_11926-model2.6 | -13.3485 |
|  | XP\_065064938\_11926-model2.7 | -13.3485 |
|  | XP\_065064938\_11926-model2.8 | -13.3485 |
|  | XP\_065064938\_11926-model2.9 | -13.3485 |
|  | XP\_065064938\_11926-model2.10 | -13.3485 |
| # cluster 3 - Size: 6 |  |  |
|  | XP\_065064938\_11926-model3 | -13.3327 |
|  | XP\_065064938\_11926-model3.1 | -13.3327 |
|  | XP\_065064938\_11926-model3.2 | -13.3103 |
|  | XP\_065064938\_11926-model3.3 | -13.2726 |
|  | XP\_065064938\_11926-model3.4 | -13.2726 |
|  | XP\_065064938\_11926-model3.5 | -13.2726 |
| # cluster 4 - Size: 6 |  |  |
|  | XP\_065064938\_11926-model4 | -13.3303 |
|  | XP\_065064938\_11926-model4.1 | -13.3303 |
|  | XP\_065064938\_11926-model4.2 | -13.3303 |
|  | XP\_065064938\_11926-model4.3 | -13.3303 |
|  | XP\_065064938\_11926-model4.4 | -13.3303 |
|  | XP\_065064938\_11926-model4.5 | -13.1973 |
| # cluster 5 - Size: 26 |  |  |
|  | XP\_065064938\_11926-model5 | -13.311 |
|  | XP\_065064938\_11926-model5.1 | -13.311 |
|  | XP\_065064938\_11926-model5.2 | -13.311 |
|  | XP\_065064938\_11926-model5.3 | -13.311 |
|  | XP\_065064938\_11926-model5.4 | -13.311 |
|  | XP\_065064938\_11926-model5.5 | -13.311 |
|  | XP\_065064938\_11926-model5.6 | -13.311 |
|  | XP\_065064938\_11926-model5.7 | -13.311 |
|  | XP\_065064938\_11926-model5.8 | -13.311 |
|  | XP\_065064938\_11926-model5.9 | -13.311 |
|  | XP\_065064938\_11926-model5.10 | -13.311 |
|  | XP\_065064938\_11926-model5.11 | -13.311 |
|  | XP\_065064938\_11926-model5.12 | -13.311 |
|  | XP\_065064938\_11926-model5.13 | -13.311 |
|  | XP\_065064938\_11926-model5.14 | -13.311 |
|  | XP\_065064938\_11926-model5.15 | -13.311 |
|  | XP\_065064938\_11926-model5.16 | -13.311 |
|  | XP\_065064938\_11926-model5.17 | -13.311 |
|  | XP\_065064938\_11926-model5.18 | -13.311 |
|  | XP\_065064938\_11926-model5.19 | -13.311 |
|  | XP\_065064938\_11926-model5.20 | -13.311 |
|  | XP\_065064938\_11926-model5.21 | -13.311 |
|  | XP\_065064938\_11926-model5.22 | -13.311 |
|  | XP\_065064938\_11926-model5.23 | -13.311 |
|  | XP\_065064938\_11926-model5.24 | -13.311 |
|  | XP\_065064938\_11926-model5.25 | -13.311 |
| # cluster 6 - Size: 6 |  |  |
|  | XP\_065064938\_11926-model6 | -13.2876 |
|  | XP\_065064938\_11926-model6.1 | -13.2876 |
|  | XP\_065064938\_11926-model6.2 | -13.2876 |
|  | XP\_065064938\_11926-model6.3 | -13.2876 |
|  | XP\_065064938\_11926-model6.4 | -13.2203 |
|  | XP\_065064938\_11926-model6.5 | -13.2203 |
| # cluster 7 - Size: 4 |  |  |
|  | XP\_065064938\_11926-model7 | -13.2857 |
|  | XP\_065064938\_11926-model7.1 | -13.2857 |
|  | XP\_065064938\_11926-model7.2 | -13.2857 |
|  | XP\_065064938\_11926-model7.3 | -13.1358 |
| # cluster 8 - Size: 6 |  |  |
|  | XP\_065064938\_11926-model8 | -13.2631 |
|  | XP\_065064938\_11926-model8.1 | -13.2631 |
|  | XP\_065064938\_11926-model8.2 | -13.2631 |
|  | XP\_065064938\_11926-model8.3 | -13.2631 |
|  | XP\_065064938\_11926-model8.4 | -13.2631 |
|  | XP\_065064938\_11926-model8.5 | -13.2631 |
| # cluster 9 - Size: 4 |  |  |
|  | XP\_065064938\_11926-model9 | -13.2416 |
|  | XP\_065064938\_11926-model9.1 | -13.2416 |
|  | XP\_065064938\_11926-model9.2 | -13.2416 |
|  | XP\_065064938\_11926-model9.3 | -13.2416 |
| # cluster 10 - Size: 3 |  |  |
|  | XP\_065064938\_11926-model10 | -13.2405 |
|  | XP\_065064938\_11926-model10.1 | -13.2405 |
|  | XP\_065064938\_11926-model10.2 | -13.2186 |
| # cluster 11 - Size: 1 |  |  |
|  | XP\_065064938\_11926-model11 | -13.2186 |
| # cluster 12 - Size: 1 |  |  |
|  | XP\_065064938\_11926-model12 | -13.1623 |
|  |  |  |
